# 

**Published:** 1881-01

**Authors:** 


### MISSISSIPPI VALLEY DENTAL SOCIETY.

The thirty-seventh annual meeting of the Mississippi Valley Dental Society will be held in the lecture room of the Ohio College of Dental Surgery, on the first Wednesday of March, 1881, at 10 o'clock, A. M., at which it is to be hoped there will be a large representation of the profession.

This will, doubtless, be an interesting occasion. It is very probable that some of the earlier members of the Society, who have been absent for several years, will be present. It is almost certain that Dr. Geo. Watt will be of that number; his health having been restored to a remarkable degree.

The proceedings of this Society are always interesting and instructive. There are other things at this time that make it an occasion of special interest The annual meeting of the Ohio Dental College Association, and the annual meeting of the Alumni Association of the College, and the College Commencement, all take place at this time.

The following programme of subjects has been presented by the Executive Committee of the Mississippi Valley Dental Society, for consideration at that meeting, viz:

1. Alveolar Abscess, and Treatment.
2. Treatment of Superficial Caries of the Teeth.
3. To what extent does Vitality resist the encroachment of Dental Caries?
4. Removal of the first permanent molars. When indicated?
5. Pathology and Treatment of Exposed Pulps.
6. Diseases of the Antrum and Treatment.

7. To what extent should other materials than gold be used in Filling Teeth ?

8. Artificial Dentures.

It is very desirable that the members should study these subjects, in order that the best thought be presented upon each.

### HYMENIAL.

MARRIED, Nov. 24th, 1880, at the residence of the bride's parents, Dr. Edward G. Betty, D. D. S., to Miss Cora B. Barrick, both of Cincinnati.

Dr. Betty, as he usually does, has in this matter done a good and proper thing. In proof of this see Prov. xviii : 22.

The happy pair will have the best wishes of all their friends for a bright and joyous future; may it grow brighter and brighter, in the ongoing of life's voyage, and new beauties and pleasures spring up in all the way.

### CONTENTS.

### COMMUNICATIONS.

A MONTHLY MAGAZINE DEVOTED TO THE INTERESTS OF

**THE DENTAL PROFESSION.**

*TERMS REDUCED TO $2.50 PER YEAR. SINGLE COPIES 25C.*

EDITED BY PROF. J. TAFT, D.D.S.

AND

*Published by SPENCER, CROCKER & CO., Ohio Dental Depot.*

The volume commences in January and closes in December.

The REGISTER being issued monthly is always fresh, therefore Indispensable to the practitioner who desires to keep posted up with the new inventions and improvements which are daily developed. Neither expense nor effort will be spared to give value and variety to its contents. Articles will be illustrated with well executed engravings whenever they will add to the interest or assist to explanation.

Its extensive circulation and frequency of issue make it one of the BEST DENTAL ADVERTISING MEDIUMS in the United States.

All subscriptions must begin with January or July.

Local or special notices, five lines or less, will be inserted for$3 each insertion; over five lines, 50 cts. per line.

All advertising bills payable in advance, or at furthest, after the issue of the first number containing the advertisement. All transient advertisements to be paid for in advance.

☞CONTRIBUTIONS SOLICITED.

Exclusively Editorial Communications should be addressed to Editor.

The latest number of the REGISTER may be ordered with or without oth-3 goods at any time, and all letters should be addressed to

*SPENCER, CROCKER & CO., Ohio Dental Depot, Cincinnati, O.*

